# Rapid monitoring of SARS-CoV-2 variants of concern through high-resolution melt analysis

**DOI:** 10.1038/s41598-023-48929-1

**Published:** 2023-12-07

**Authors:** Aurora Diotallevi, Gloria Buffi, Simone Barocci, Marcello Ceccarelli, Daniela Bencardino, Francesca Andreoni, Chiara Orlandi, Marilisa Ferri, Daniela Vandini, Stefano Menzo, Eugenio Carlotti, Anna Casabianca, Mauro Magnani, Luca Galluzzi

**Affiliations:** 1 Section of Biotechnology, Department of Biomolecular Sciences, University of Urbino Carlo Bo, 60132 Fano, PU Italy; 2 Department of Clinical Pathology, Azienda Sanitaria Territoriale (AST) Pesaro e Urbino, Marche, 61029 Urbino, PU Italy; 3 Virology Laboratory, Azienda Ospedaliero Universitaria delle Marche, 60126 Ancona, AN Italy; 4 Department of Prevention, Azienda Sanitaria Territoriale (AST) Pesaro e Urbino Marche, 61029 Urbino, PU Italy

**Keywords:** Biological techniques, Biotechnology, Molecular biology

## Abstract

The current global pandemic of COVID-19 is characterized by waves of infection due to the emergence of new SARS-CoV-2 variants carrying mutations on the Spike (S) protein gene. Since autumn 2020 many Variants of Concern (VOC) have been reported: Alpha/B.1.1.7, Beta/B.1.351, Gamma/P.1, Delta/B.1.617.2, Omicron/B.1.1.529, and sublineages. Surveillance of genomic variants is currently based on whole-genome sequencing (WGS) of viral genomes on a random fraction of samples positive to molecular tests. WGS involves high costs, extended analysis time, specialized staff, and expensive instruments compared to a PCR-based test. To rapidly identify the VOCs in positive samples, six assays based on real-time PCR and high-resolution melting (HRM) were designed on the S gene and applied to 120 oro/nasopharyngeal swab samples collected from October 2020 to June 2022 (106 positive and 14 negative samples). Overall, the assays showed 100% specificity and sensitivity compared with commercial PCR tests for COVID-19. Moreover, 104 samples out of 106 (98.1%) were correctly identified as follows: 8 Wuhan (wild type), 12 Alpha, 23 Delta, 46 Omicron BA.1/BA.1.1, 15 Omicron BA.2/BA.4/BA.5. With our lab equipment, about 10 samples can be processed every 3 h at the cost of less than € 10 ($ 10.60) per sample, including RNA extraction. The implementation of this approach could help local epidemiological surveillance and clinical decision-making.

## Introduction

RNA viruses, including SARS-CoV-2, the causative agent of the twenty-first-century COVID-19 pandemic, constantly evolve through genome modifications. Most changes have little or no impact on the virus phenotype. However, a small minority of mutations can confer a selective advantage and new features to the virus in terms of increased transmissibility^1^, vaccine response^2,3^, pathogenicity or greater severity of the associated disease^4^, and response to monoclonal antibody therapy^5^. Therefore, rapid detection and identification of SARS-CoV-2 variants and monitoring of their prevalence are critical and necessary.

Since September 2020, new variants of the original strain of SARS-CoV-2 have emerged that have caused an increased risk to global public health. According to the World Health Organization, emerging variants have been classified based on specific parameters, such as the severity of the disease and the global distribution, in Variants Of Interest (VOIs) and Variants Of Concern (VOCs), considered more dangerous, in order to monitor and identify the most correct strategy to be adopted in the fight to the COVID-19 pandemic^6^. In addition to these, Variants Under Monitoring (VUMs) include variants of SARS-CoV-2 with mutations suspected to affect the characteristics of the virus, but the phenotypic or epidemiological impact is currently unclear and requires further monitoring^6^.

Concerning VOCs, Alpha (B.1.1.7 and sublineages), Beta (B.1.351), Gamma (P.1 and sublineages), Delta (B.1.617.2 and sublineages), and Omicron (B.1.1.529 and sublineages) have been reported. At the time we drafted this manuscript, the Omicron sublineages BA.2, BA.3, BA.4, and BA.5 were responsible for COVID-19 new cases, as reported by CoVariants^7^.

Currently, whole-genome sequencing (WGS) is employed for the characterization of SARS-CoV-2 variants worldwide, and millions of sequences have been recorded in public databases, such as the Global Initiative on Sharing All Influenza Data (GISAID)^8^ or GenBank. The WGS not only requires a long time of processing and professional expertise for data analysis, but it is also expensive in terms of equipment and reagents. Reverse transcription real-time PCR (RT-qPCR) could be a rapid method for the identification of VOCs^9^. In particular, an approach based on real-time PCR coupled with high-resolution melting (HRM) analysis could be considered a faster and cheaper alternative to the WGS^10,11^. In fact, in the last decades, the HRM analysis employing saturating or non-saturating DNA intercalating dyes has gained increasing interest due to its ability to monitor mutations and genotyping in many research fields, such as epidemiology and microbiology. For example, the use of HRM analysis has previously been widely applied for detecting bacteria and antimicrobial resistance genes^12,13^, genotyping protozoan parasites such as *Plasmodium falciparum*^14^ and *Leishmania* spp.^15,16^, testing drug susceptibility in influenza A viruses^17^, testing drug resistance in hepatitis B virus^18^, and studying HIV diversity^19^.

Concerning SARS-CoV-2, previous works reported the detection of single nucleotide variations in the Spike protein-coding region by post-PCR HRM analysis. For instance, Gazali et al.^20^ and Aoki et al.^11^ reported the detection of D614G (nucleotide mutation: A23403G) and L452R (nucleotide mutation: T22917G) variations, respectively. Moreover, HRM analysis has been used to detect SARS-CoV-2 Omicron (B.1.1.529)-specific mutations G339D (Nucleotide mutation: G22578A) and D796Y (Nucleotide mutation: G23948T)^21^.

In this work, we describe the development and assessment of a rapid, cost-effective method based on HRM analysis to detect SARS-CoV-2 VOCs in a routine clinical setting. The SARS-CoV-2 Spike protein-coding region PCR amplicons are identified by unique mutation signatures, such as single nucleotide polymorphisms (SNPs) and deletions, with the aim to detect VOCs without the need for labeled probes. The method relies on the use of a versatile diagnostic algorithm that could be adapted depending on circulating variants.

## Results

### Selection of SARS-CoV-2 mutations in the S protein gene

Sequence comparison analysis of S gene allowed identifying six regions containing sequence polymorphisms (SNPs and/or deletions) characterizing the different VOCs (Alpha, Beta, Gamma, Delta, Omicron BA.1, Omicron BA.1.1, Omicron BA.2, Omicron BA.3, Omicron BA.4, and Omicron BA.5) (Table 1). Briefly, the polymorphisms in the S gene identified for designing the qPCR-HRM assays for each VOC were: C1709A (Alpha); A644G and del721/729 (Beta); C3080T (Gamma); G460A and del467/472 (Delta); C2568A (Omicron BA.1, Omicron BA.1.1); G425A and del425/433 (Omicron BA.2, Omicron BA.3, Omicron BA.4, and Omicron BA.5). All selected polymorphisms induced mutation on the amino acid sequence. Six qPCR-HRM assays were designed on these regions, with the aim to exploit the difference in amplicon melting temperatures to differentiate each VOC. The selected mutations were confirmed to be present in the VOCs using Lineage comparison from outbreak.info (https://outbreak.info/compare-lineages) (Supplementary Fig. 1)^22,23^.

**Table 1:** Selected mutations characterizing each VOC.

Mutation (aa)	Mutation (nucleotide)*	SARS-CoV-2 VOCs							
		Alpha	Beta	Gamma	Delta	Omicron BA.1 BA.1.1	Omicron BA.2	Omicron BA.3 BA.3.1	Omicron BA.4 BA.5
A570D	C1709A	✓							
D215G del241/243	A644G del721/729		✓						
S982A	T2944G	✓							
T1027I	C3080T			✓					
E156G del157/158	del467/472				✓				
N856K	C2568A					✓			
G142D	G425A						✓		✓
G142D del143/145	del425/433					✓		✓	

*Referred to the reference strain Wuhan-H-1 S gene sequence (accession number NC_045512.2, nucleotides: 21,563–25,384).

### Evaluation of RT-qPCR assays sensitivity and specificity

Primer specificity was first checked in silico either with human genome or with common human coronaviruses (i.e., Alpha coronavirus 229E, NL63; Beta coronavirus OC43, HKU1). After amplification from oro/nasopharyngeal swab samples, selected PCR products were analyzed by agarose gel electrophoresis showing bands at the expected size and the absence of non-specific products or primer dimers (Supplementary Fig. 2). All positive and negative results obtained with commercial IVD-certified molecular tests were confirmed with the qPCR assays described in this paper, accounting for 100% specificity and sensitivity of our assays. The Ct values in our assays were < 30 in 105 out of 106 positive samples. The analytical sensitivity was tested using SARS-CoV2 synthetic RNA of the reference isolate Wuhan-hu-1 as described in methods. The linear limit of detection of all assays was 1 × 10^2^ viral genome copies/reaction tube (with or without background host RNA), except for assay 2930–3100, which showed a sensitivity of 1 × 10^3^ viral genome copies/reaction tube (Supplementary Fig. 3).

Moreover, all assays were tested by qPCR to evaluate the specificity using RNA from 10 clinical specimens positive for common respiratory viruses and 4 DNA samples extracted from colonies of respiratory pathogen bacteria, as described in methods. PCR mixtures were analyzed by agarose gel electrophoresis, confirming the absence of cross-reactivity with these pathogens (Supplementary Fig. 4). The amplifiability of RNA from clinical specimens positive for other respiratory viruses was confirmed using a commercial IVD-certified kit for differential diagnosis of infections by SARS-CoV-2, Influenza A, Influenza B and Respiratory Syncytial Virus A/B, which included RNase P gene as endogenous control. The results confirmed the positivity of those samples and their amplifiability (Supplementary Table 1).

### SARS-CoV-2 VOCs discrimination

A total of 120 oro/nasopharyngeal swab samples collected from October 2020 to June 2022 (106 positive samples and 14 negative samples) were used to validate our assays.

**Figure 1:**
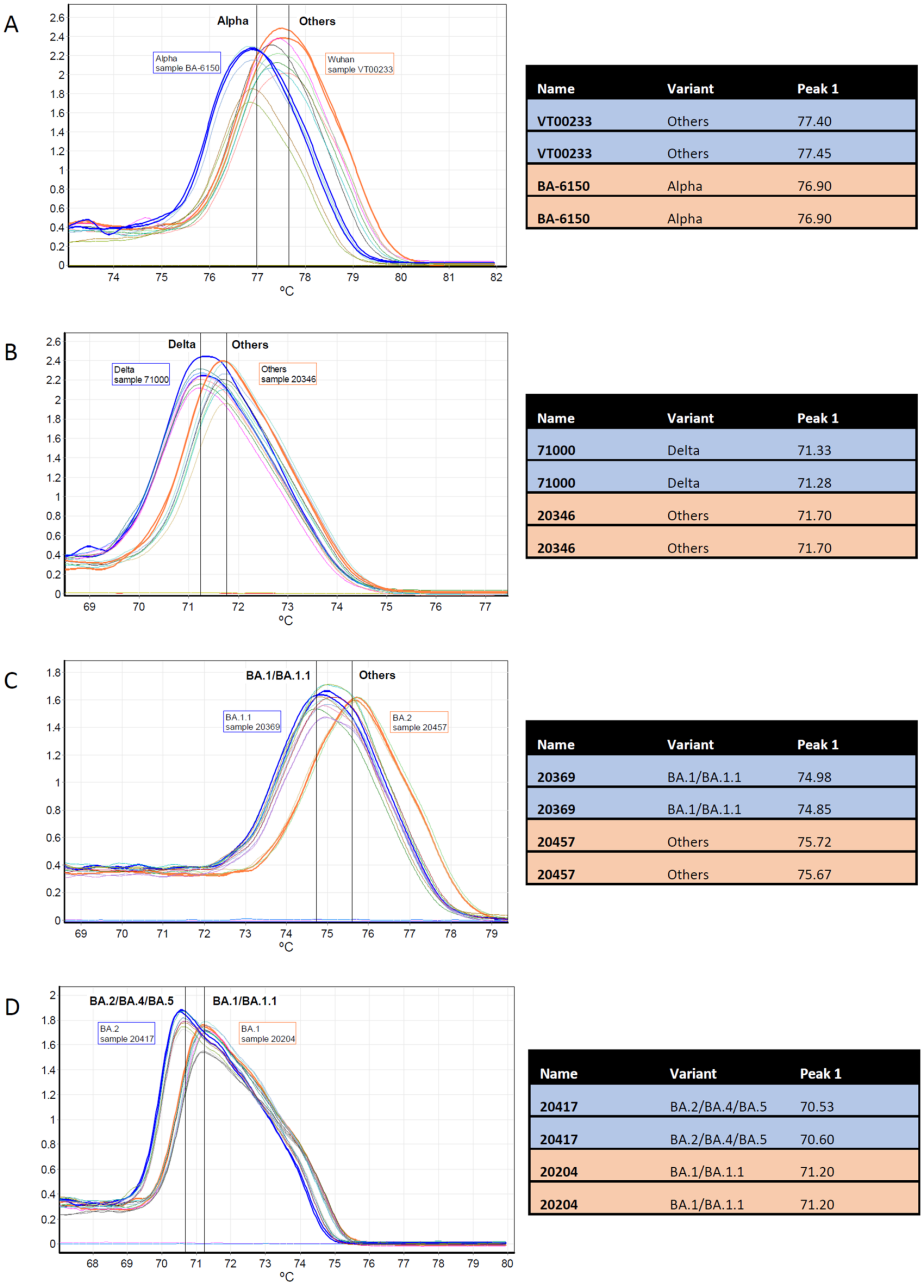
Representative HRM profiles of qPCR assay 1710 (**A**), SC2DELTAnew (**B**), OMICRON CA (**C**), and DEL9 (**D**). The plot of the negative derivative of fluorescence (dF/dT) vs temperature is shown, and the melting transitions are represented as peaks. Reference samples are evidenced as bold curves, while the negative controls (no template controls) are represented as flat curves. The amplicons containing/not containing the mutation were clearly distinguishable. Melting profiles in panels (**A**–**D**) were obtained with one-step RT-qPCR mix. Each sample was tested in duplicate.

Each of the following qPCR assays was useful to discriminate specific mutations from the others: the qPCR assay 1710 discriminated the Alpha variant (carrying the mutation A570D; Fig. 1A); the qPCR assay SC2DELTAnew discriminated Delta variant (carrying the mutation E156G and F157_R158del; Fig. 1B); the qPCR assay OMICRON CA discriminated omicron BA.1/BA.1.1 variants (carrying the mutation N856K; Fig. 1C). Instead, the qPCR assay DEL9 discriminated omicron BA.1/BA.1.1, BA.3 from BA.2, BA.4, BA.5 (Fig. 1D). The qPCR assay 1710 generated amplicons having HRM temperature of 76.98 ± 0.18 °C for the Alpha variant and 77.65 ± 0.30 °C for the other variants (RT-qPCR mix Qiagen); the assay SC2DELTAnew produced amplicons with distinct HRM temperatures for the Delta variant compared to the other variants, either using the RT-qPCR mix Qiagen (71.24 ± 0.10 °C and 71.76 ± 0.11 °C, respectively) or Takara (75.27 ± 0.13 °C and 75.66 ± 0.08 °C, respectively); the assay OMICRON CA produced amplicons with distinct HRM temperatures for the Omicron BA.1/BA.1.1 variants compared to all others, either using the RT-qPCR mix Qiagen (74.73 ± 0.14 °C and 75.41 ± 0.21 °C, respectively) or Takara (78.67 ± 0.10 °C and 79.29 ± 0.18 °C, respectively); finally, the assay DEL9 discriminated within the Omicron variants, between the sublineages BA.1/BA.1.1, BA3 and BA.2, BA.4, BA5, using the RT-qPCR mix Qiagen (71.24 ± 0.07 °C and 70.82 ± 0.22 °C, respectively) and Takara (76.18 ± 0.05 °C and 75.80 ± 0.07 °C, respectively). Average values and SD of high-resolution melting temperatures for each amplicon obtained with RT-qPCR mix (Qiagen) and qPCR mix (Takara) are summarized in Tables 2 and 3, respectively. Since samples containing VOCs Beta and Gamma were not available, the discriminatory capability of the assays 645–770 and 2930–3100 was not experimentally tested.

**Table 2:** Average values and SD of HRM temperatures for each amplicon obtained with RT-qPCR mix Qiagen.

qPCR assay_VOC	Mean HRM temperature (°C)	SD	N of tested samples	N of replicates	95% CI	p value*
1710_Alpha	76.98	0.18	12	24	76.87–77.09	Two-tailed p < 0.0001
1710_others	77.65	0.30	18	36	77.50–77.80	
645-770_Beta	–	–	–	–	–	–
645-770_others	76.52	0.07	4	8	76.41–76.63	
2930-3100_Gamma	–	–	–	–	–	–
2930-3100_others	77.88	0.10	3	6	77.78–77.98	
SC2DELTAnew_Delta	71.24	0.10	11	22	71.17–71.31	Two-tailed p < 0.0001
SC2DELTAnew_others	71.76	0.11	30	60	71.72–71.80	
CA_BA.1/BA.1.1	74.73	0.14	38	76	74.68–74.78	Two-tailed p < 0.0001
CA_others	75.41	0.21	17	34	75.30–75.52	
DEL9_BA.1/BA.1.1, BA3	71.24	0.07	6	12	71.21–71.35	Two-tailed p < 0.0004
DEL9_BA.2, BA.4, BA5	70.82	0.22	8	16	70.64–71.00	

*Unpaired *t*-test with Welch’s correction.

**Table 3:** Average values and SD of HRM temperatures for each amplicon obtained with RT-qPCR mix Takara.

qPCR assay_VOC	Mean HRM temperature (°C)	SD	N of tested samples	N of replicates	95% CI	p value*
SC2DELTAnew_Delta	75.27	0.13	10	20	75.18–75.36	Two-tailed p < 0.0001
SC2DELTAnew_others	75.66	0.08	23	46	75.63–75.69	
CA_BA.1/BA.1.1	78.67	0.10	19	42	78.62–78.72	Two-tailed p < 0.0001
CA_others	79.29	0.18	36	74	79.23–79.35	
DEL9_BA.1/BA.1.1, BA.3	76.18	0.05	8	16	76.14–76.22	Two-tailed p < 0.0001
DEL9_BA.2, BA.4, BA.5	75.80	0.07	13	26	75.76–75.84	

*Unpaired *t*-test with Welch’s correction.

**Figure 2:**
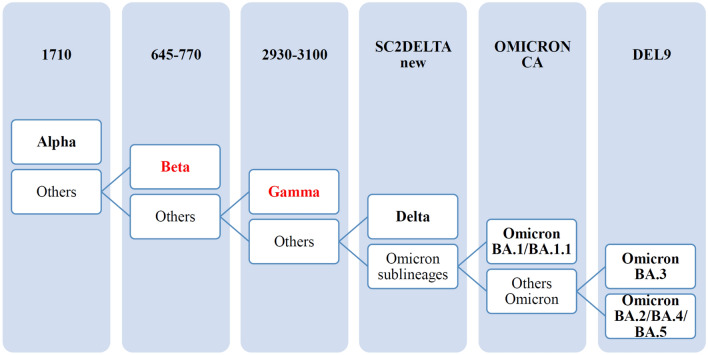
Hierarchical approach based on qPCR and HRM analysis targeting variable regions in the Spike gene for identification of SARS-CoV-2 VOCs. The names of qPCR assays are boxed. Samples containing VOCs Beta and Gamma (in red) were not available.

These assays can be performed hierarchically in a diagnostic algorithm (Fig. 2). Through this approach we were able to discriminate the main VOCs circulating by the time of writing this manuscript. For example, within the Omicron variants, the hierarchical approach “OMICRON CA” followed by “DEL9” allows to distinguish the three clusters [BA.1/BA.1.1], [BA.3] and [BA.2/BA.4/BA.5]. The results were confirmed by PCR product sequencing and/or by the available WGS data. Positive percent agreement (sensitivity) and negative percent agreement (specificity) for each assay, using sequencing as the reference method, are reported in Table 4.

**Table 4:** Comparison of HRM analysis and sequencing results (WGS or amplicon sequencing) for SARS-CoV-2 Spike gene mutation detection.

Nucleotide mutations and HRM result	Sequence result		Sensitivity (%) (95% CI)	Specificity (%) (95% CI)
	Mutated	Non-mutated		
C1709A				
Mutated	12	0	100 (73.5–100)	100 (79.4–100)
Non-mutated	0	16		
A644G; del721/729				
Mutated	0	0	–	100 (39.8–100)
Non-mutated	0	4		
T2944G; C3080T				
Mutated	0	0	–	100 (29.2–100)
Non-mutated	0	3		
del467/472				
Mutated	17	0	100 (80.5–100)	100 (90.5–100)
Non-mutated	0	37		
C2568A				
Mutated	32	1	100 (89.1–100)	96.5 (82.2–99.9)
Non-mutated	0	28		
G425A; del425/433				
Mutated	8	1	100 (63.1–100)	93.3 (68.1–99.8)
Non-mutated	0	14		

All data are resumed in Supplementary Table 2. In summary, the qPCR assays followed by HRM analysis allowed to assign the correct VOC in all positive samples, with the exception of two samples (20044, 49095). Sample 20044 was tested with two qPCR assays (SC2DELTAnew and OMICRON CA), but in this case, the HRM profiles did not allow to assign a genotype. In fact, the PCR products sequencing showed the presence of two possible amplicons: with and without 467/472 deletion, and with and without C2568A mutation for qPCR assay SC2DELTAnew and OMICRON CA, respectively (Supplementary Fig. 5). Sample 49095 was tested with qPCR assays OMICRON CA and DEL9. The HRM analysis assigned this sample to Omicron BA.1/BA.1.1 variant; however, this result did not match with WGS data that indicated Omicron BA.4 variant.

## Discussion

As of Autumn 2020, several countries have reported the detection of SARS-CoV-2 variants, characterized by high transmissibility or reduced susceptibility to neutralizing antibodies induced by infection or vaccination^5^. The genome of these variants presents a number of mutations and some are included in the ACE2 binding domain of the Spike protein, increasing the viral binding to host cells^24–27^. Surveillance of genomic variants, together with compliance with public health measures (vaccination, use of masks, isolation, and quarantine), has been essential to limit the spread of SARS-CoV-2.

Currently, the surveillance of genomic variants is based on the WGS of viral genomes, able to explore the whole SARS-CoV-2 genome in detail^28,29^ even though it is performed on a minority fraction of positive samples. However, WGS approaches are characterized by high costs and extended analysis times compared to PCR-based diagnostic tests, and the delay in obtaining WGS results could hinder the public health response and real-time prevalence evaluation of the different variants in the population. As an alternative to WGS, other methods for variant identification have been reported. Very recently, Burgos et al. proposed a method based on PCR amplification of four polymorphic genetic regions coupled with Sanger sequencing^30^. Although this approach has lower costs and time of processing compared to WGS, it is still time-consuming, and it cannot detect new mutations in a region of the viral genome different from those considered for sequencing. Moreover, it requires expensive instrumentation (Sanger sequencing instrument). Among the molecular approaches attempted to simplify the typing process and lower its cost, several qPCR methods based on probes targeting different SARS-CoV-2 mutations have been introduced. For example, Yeung et al*.* designed and validated a multiplex RT-qPCR assay based on labeled probes targeting Spike protein mutations to detect Alpha, Beta, Gamma, Delta, and Omicron VOCs^31^. The probes increase the specificity of the assay but their use can be expensive and could give false negative results in case of mismatches to the targeted site^32^. For these reasons, researchers have started to develop faster, cheaper, and more flexible tests which can be adapted as needed. Our study is centered on the development of a qPCR coupled with HRM analysis for SARS-CoV-2 variants identification. HRM analysis can be used to assess whether two or more PCR products of similar size, amplified by the same primer pair, have identical nucleotide sequence. After the amplification, the PCR products are subjected to small increases in temperature (generally 0.1–0.3 °C for 2–10 s) to reach the melting temperature to which the double-stranded amplicon is denatured. Therefore, based on Tm differences, it is possible to determine the presence of mutations (SNPs and indels). This approach is more affordable than a probe-based approach and it has been used widely for typing pathogens, including RNA viruses^33–35^.

In the attempt to develop an economical tool for the rapid screening of the main SARS-CoV-2 VOCs, we designed a diagnostic algorithm using six different hierarchical qPCR-HRM assays able to discriminate the main VOCs circulating from the beginning of the pandemic until the time we drafted this manuscript. The choice and the number of assay(s) will be decided by the operator and will be based on the current epidemiological situation. As new variants emerge, the diagnostic algorithm could be updated with novel designed assays.

This approach has proven to be very effective, showing 97.7% agreement with sequencing data (84 out of 86 samples with sequence information). Only samples 20044 and 49095 displayed HRM results that were not confirmed by sequencing data. Concerning sample 20044, as described in the results, the PCR product sequences showed the presence of two amplicons corresponding to Delta and Omicron BA.1/BA.1.1 variants, indicating a possible co-infection, as previously reported^36^. Moreover, it is noteworthy that this sample was collected on December 19th, 2021, when Delta and Omicron variants were co-existing in our territory. Concerning sample 49095, the Ct values of the two qPCR assays used for variant identification (OMICRON CA and DEL9) were both > 30; this delay in amplification could have affected the reliability of HRM analysis, as reported previously^37–39^.

The qPCR-HRM assays were tested using two commercial mixtures (one-step Qiagen and two-step Takara), both showing the ability to differentiate mutated from unmutated amplicons. While the one-step approach allows to slightly shorten the time of analysis, the two-step approach allows for long-term storage of cDNA samples and increases reproducibility over time, since low-concentration RNA samples may not produce consistent results after freeze–thaw cycles^31^.

Concerning the timing and costs, with our laboratory equipment, it is possible to process up to 10 samples every 3 h, starting from the RNA extraction, with a cost of less than € 10 ($ 10.60) per sample. In addition, the choice of a qPCR mixture containing SYBR green dye instead of using a saturating dye (e.g. Eva Green, SYTO9, or LC Green) makes this approach more affordable, also in the perspective of a large-scale screening method. In fact, it has been previously demonstrated that using the Rotor-Gene 6000 instrument, HRM analysis results were effective also using SYBR Green^38,40^. Therefore, this method can be considered a cost-effective approach for the fast screening of VOCs with the aim of facilitating local epidemiological surveillance and limiting the number of samples subjected to WGS.

Nevertheless, the HRM-based approach has some limitations. First, it is important to note that such typing assays may provide atypical results for emerging variants due to new mutations within the primer binding sites, resulting in a decrease in amplification efficiency, and/or between the primers, resulting in a less effective variant recognition. In this case, confirmation or deeper analysis can be performed in selected samples (where the variant could not be identified) using established WGS approaches, allowing for more targeted use of WGS in epidemiological surveillance of new circulating variants, therefore reducing time and costs for analysis. As new variants emerge, new assays should be designed and optimized before being included in the diagnostic algorithm. Moreover, it is needed to take into account that inconclusive or low-resolution HRM data can be obtained with a poor amplification curve showing Ct > 30 or failing to reach a plateau in the PCR phase^37,38^. To partially overcome this issue, Promja et al. have developed an automated machine-learning web application capable of identifying SARS-CoV-2 variants by interpreting HRM profiles^41^. The authors set the Ct threshold value for variant identification at 33.4, allowing to include samples with low amounts of viral RNA. Finally, it is worth mentioning that the applicability of our method with other qPCR mixtures and instruments different from those used in this work must be optimized, and internal control to establish the HRM range for each assay needs to be included.

In summary, this approach could be beneficial in terms of time and costs since it is characterized by modest reagent requirements and utilizes instrumentation already present in many routine clinical and public health laboratories. The development of qPCR-HRM assays has the potential to genetically characterize SARS-CoV-2 VOCs and can be an alternative or an important complement to WGS-based epidemiological surveillance that can directly impact the clinical care of individual patients. Since our approach demonstrated 100% specificity and sensitivity compared with commercial PCR tests for COVID-19, it could be used either to find SARS-CoV-2 positive patients or to monitor already known SARS-CoV-2 variants for epidemiological purposes. Nevertheless, in case of the emergence of new variants, sequencing-based approaches will still be needed to identify new mutations and to allow the design of new qPCR assays.

## Material and methods

### Ethical statement

The study was conducted in accordance with the Declaration of Helsinki and approved by the Ethics Committee (Comitato Etico per la Sperimentazione Umana, CESU) of the University of Urbino Carlo Bo (protocol number n. 46/2022). The approved study protocol included the informed consent forms for the subjects involved.

### Identification of polymorphic sites and primer design

Sequences of SARS-CoV-2 reported from countries around the world were randomly downloaded from the EpiCoV database of Global Initiative on Sharing All Influenza Data (GISAID)^8^, the most complete repository of coronavirus-causing COVID-19 genomic data. Selected sequences of each VOC were downloaded from GISAID, loaded on Jalview^42^, and multiple sequence alignment (MSA) was performed using the Multiple Sequence Comparison by Log-Expectation (MUSCLE)^43^. All MSA for each variant was performed against the reference strain Wuhan-H-1 sequence (NCBI GenBank accession number NC_045512.2). For our purpose, only the Spike-encoding region was considered. Among all polymorphisms, those conferring changes in theoretical melting temperatures (Tm) (at least 0.3 °C), potentially allowing the variants discrimination, were identified (Table 1). Amplicon theoretical melting temperatures were determined using Bioedit Sequence Alignment Editor 7.2.5^44^. Primers upstream and downstream of these mutations were designed and checked for specificity using Primer-BLAST^45^ (Table 5).

**Table 5:** qPCR assays and primer sequences encompassing target mutations.

Mutation target (nucleotide)*	qPCR assay name	Primer sequence (5′–3′)	PCR product length (bp)
C1709A	1710	F: ACAGGCACAGGTGTTCTTACT R: CTGTGGATCACGGACAGCAT	102
A644G del721/729	645–770	F: GCACACGCCTATTAATTTAGTGC R: AGCTGTCCAACCTGAAGAAGA	154–163
T2944G C3080T	2930–3100	F: TTTTGGTGCAATTTCAAGTGTTTT R: TTTGATTGTCCAAGTACACACTCT	207
del467/472	SC2DELTAnew	F: AACAACAAAAGTTGGATGGAAAGTG R: CTGAGAGACATATTCAAAAGTGCAA	72–78
C2568A	OMICRON CA	F: TGCTGCTAGAGACCTCATTTGT R: ATCTGTGAGCAAAGGTGGCAA	70
G425A del425/433	DEL9	F: ACCCAGTCCCTACTTATTGTTAAT R: ACTTTCCATCCAACTTTTGTTGTT	117–126

*Referred to the reference strain Wuhan-H-1 S gene sequence (accession number NC_045512.2, nucleotides: 21,563–25,384).

### Sample collection

All samples used in this study were surplus material collected for diagnostic purposes during routine examinations. Oro/Nasopharyngeal swabs samples collected in viral transport medium were obtained from Urbino Hospital (ASUR Marche AV1)-Laboratory of Clinical Pathology (Urbino, Italy), Covid-Lab (University of Urbino, Fano, Italy) and Virology Laboratory, Azienda Ospedaliera Ospedali Riuniti di Ancona (Ancona, Italy). A total of 120 samples tested with IVD-certified RT-qPCR kits (Diatheva COVID-19 PCR Kit, Diasorin Simplexa™ COVID-19 Direct Kit, Seegene Allplex™ 2019-nCoV Assay) were selected for the study (106 positive samples and 14 negative samples). The positive samples had a Ct < 30 for all viral target sequences. The VOCs were previously identified in part of these samples through WGS approaches.

In addition, clinical specimens positive for common respiratory viruses were obtained from Virology Laboratory, Azienda Ospedaliera Ospedali Riuniti di Ancona (Ancona, Italy). These samples are detailed as follows: influenza A/H1N1 (two clinical samples), influenza A/H3N2 (two clinical samples), influenza B/Victoria (two clinical samples), respiratory syncytial virus A (two clinical samples), respiratory syncytial virus B (two clinical samples). Moreover, DNA extracted from cultivated respiratory pathogen bacteria (i.e., *Klebsiella pneumoniae* ATCC 27736, *Klebsiella pneumoniae* ATCC 700603*, Staphylococcus aureus* ATCC 29213 and *Pseudomonas aeruginosa* ATCC 15692) were kindly provided by Prof. Emanuela Frangipani.

### RNA purification

RNA from oro/nasopharyngeal swabs was isolated using the Total RNA Purification Kit (Norgen Biotek Corp., Thorold, ON Canada) starting from 250 μl of viral transport media following the Supplementary Protocol for Norgen’s Saliva RNA Collection and Preservation Device.

### RT-qPCR assays

Two approaches were tested for RT-qPCR: a one-step approach (reverse transcription and PCR amplification in the same tube) and a two-step approach (cDNA synthesis followed by PCR amplification), as described below. In both approaches, at least one sample previously characterized by WGS was always included as internal reference and processed in parallel to the unknown samples.

For the one-step RT-qPCR assays, 5 μl of extracted RNA were added to 35 μl of the reaction mixture containing QuantiNova SYBR Green RT-PCR Master Mix together with QuantiNova SYBR Green RT Mix (QIAGEN, Hilden, Germany) and 400 nM primers. The RT-qPCR reactions were carried out in duplicate in a final volume of 20 µl in a Rotor-Gene 6000 instrument (Corbett Life Science, Mortlake, Australia). The RT step was performed at 50 °C for 10 min followed by a PCR activation step at 95 °C for 2 min and by 40 cycles of amplification (95 °C for 5 s and 60 °C for 20 s). A melting curve analysis at the end of each run from 67–88 °C with a slope of 1 °C/s and 5 s at each temperature was conducted.

For the two-step RT-qPCR assays, the reverse-transcription reaction was prepared from 8 μl of total RNA, using the PrimeScript™ RT Master Mix (Perfect Real Time) (Takara, Kusatsu, Shiga, Japan) according to the manufacturer's instructions. The cDNA synthesis was carried out in a thermal cycler at the following temperatures: 37 °C for 15 min, and 85 °C for 10 s. At the end of the retro-transcription protocol, each sample was diluted 1:2 with RNase-free water. The qPCR was performed using 2 μl of cDNA as template in 38 μl of the reaction mixture containing the TB Green premix ex TaqII Mastermix (Takara Bio Europe, France) and 200 nM primers. The amplification reactions were carried out in duplicate in a final volume of 20 µl in a Rotor-Gene 6000 instrument (Corbett Life Science, Mortlake, Australia), with the same amplification and melting protocols described above.

In addition, in both assays, a duplicate non-template control as negative control was always present. Finally, selected PCR products were analyzed by electrophoresis in a 2.5% agarose gel to evaluate the specificity of amplification and the absence of non-specific products and/or primer dimers.

Assay specificity was experimentally evaluated with clinical specimens of common respiratory pathogens (i.e., Influenza A, Influenza B and RSV A/B). The presence of influenza and RSV viruses in these samples was assessed using the commercial IVD-certified COVID-FLU-RSV RT PCR Detection kit (Diatheva s.r.l., Fano, Italy), following manufacturer’s instructions. The kit included an endogenous control (RNase P gene) to monitor the RNA extraction process and the presence of PCR inhibitors.

### High-resolution melt (HRM) analysis

The amplicons obtained from one-step or two-step RT-qPCR assays were further subjected to HRM analysis on a Rotor-Gene 6000 instrument. Briefly, HRM was carried out over the range from 67 to 83 °C (for one-step assays) or from 71 to 84 °C (for two-step assays), rising at 0.1 °C/s and waiting for 2 s at each temperature. The gain was optimized before melting on all tubes. For each variant, bins were set to determine the Tm of amplicons. Automated classification of variant of unknown samples was performed by the Rotor-Gene software according to the presence of a derivative peak located within a defined temperature bin.

### Analytical sensitivity of the RT-qPCR assays

To evaluate the analytical sensitivity of all primer pairs, we performed one-step RT-qPCR using serial dilutions (from 1 × 10^6^ to 1 × 10^2^ viral genomes per reaction tube) of SARS-CoV2 synthetic RNA of the reference isolate Wuhan-hu-1 (MN908947.3) (Twist Bioscience, CA, USA). Moreover, to evaluate the potential interference of host RNA as background, 25 ng of human RNA were spiked into each qPCR reaction tube. The curves were obtained from two independent experiments performed in duplicate.

### PCR product sequencing

To confirm variant call in samples without WGS data, the PCR products were purified using the MinElute PCR purification kit (Qiagen) and directly sequenced, using both forward and reverse primers, as previously described^46^. The DNA sequencing was performed using the BigDye Terminator v. 1.1 Cycle Sequencing Kit on ABI PRISM 310 Genetic Analyzer (Applied Biosystems, Foster City, CA, USA). Sequences were manually edited, and nucleotide composition was compared with VOCs reference sequences using Bioedit Sequence Alignment Editor 7.2.5.

### Statistical analysis

Statistical analysis was performed using the Unpaired *t*-test with Welch's correction (Two-tailed p-value) test on GraphPad InStat (GraphPad Software, San Diego, CA). Sensitivity and specificity of the qPCR assays, including 95% CI were calculated using contingency tables (MedCalc statistical software)^47^. Values are expressed as mean ± standard deviation (SD).

### Supplementary Information

Supplementary Information.

## Data Availability

The datasets used and/or analyzed during the current study are included in the article/Supplementary Material. Further data are also available from the corresponding author on reasonable request.
