# Supplementary material for: Rapid monitoring of SARS-CoV-2 variants of concern through high-resolution melt analysis
Source: Sci Rep. 2023 Dec 7;13:21598. doi: 10.1038/s41598-023-48929-1 (PMC10703772; doi:10.1038/s41598-023-48929-1)
Supplement: Supplementary file 1 — Supplementary Information. [file 41598_2023_48929_MOESM1_ESM.docx]

**Supplementary Information**

Rapid monitoring of SARS-CoV-2 Variants of Concern through High-Resolution Melt analysis

Aurora Diotallevi^1*^, Gloria Buffi^1^, Simone Barocci^2^, Marcello Ceccarelli^1,2^, Daniela Bencardino^1^, Francesca Andreoni^1,2^, Chiara Orlandi^1^, Marilisa Ferri^2^, Daniela Vandini^2^, Stefano Menzo^3^, Eugenio Carlotti^4^, Anna Casabianca^1^, Mauro Magnani^1^, Luca Galluzzi^1^

**Authors’ affiliation**

^1^Biotechnology Unit, Department of Biomolecular Sciences, University of Urbino Carlo Bo, Fano, PU, Italy

^2^Department of Clinical Pathology, Azienda Sanitaria Territoriale (AST) Pesaro e Urbino, Marche, 61029 Urbino, PU, Italy

^3^Virology Laboratory, Azienda Ospedaliero Universitaria delle Marche, Ancona, AN, 60126, Italy

^4^Department of Prevention, Azienda Sanitaria Territoriale (AST) Pesaro e Urbino Marche, 61029 Urbino, PU, Italy

*** Corresponding Author:**Aurora Diotallevi –

Section of Biotechnology, Department of Biomolecular Sciences, University of Urbino Carlo Bo, Via Arco d’Augusto 2 – 60132 Fano, PU, Italy

aurora.diotallevi@uniurb.it


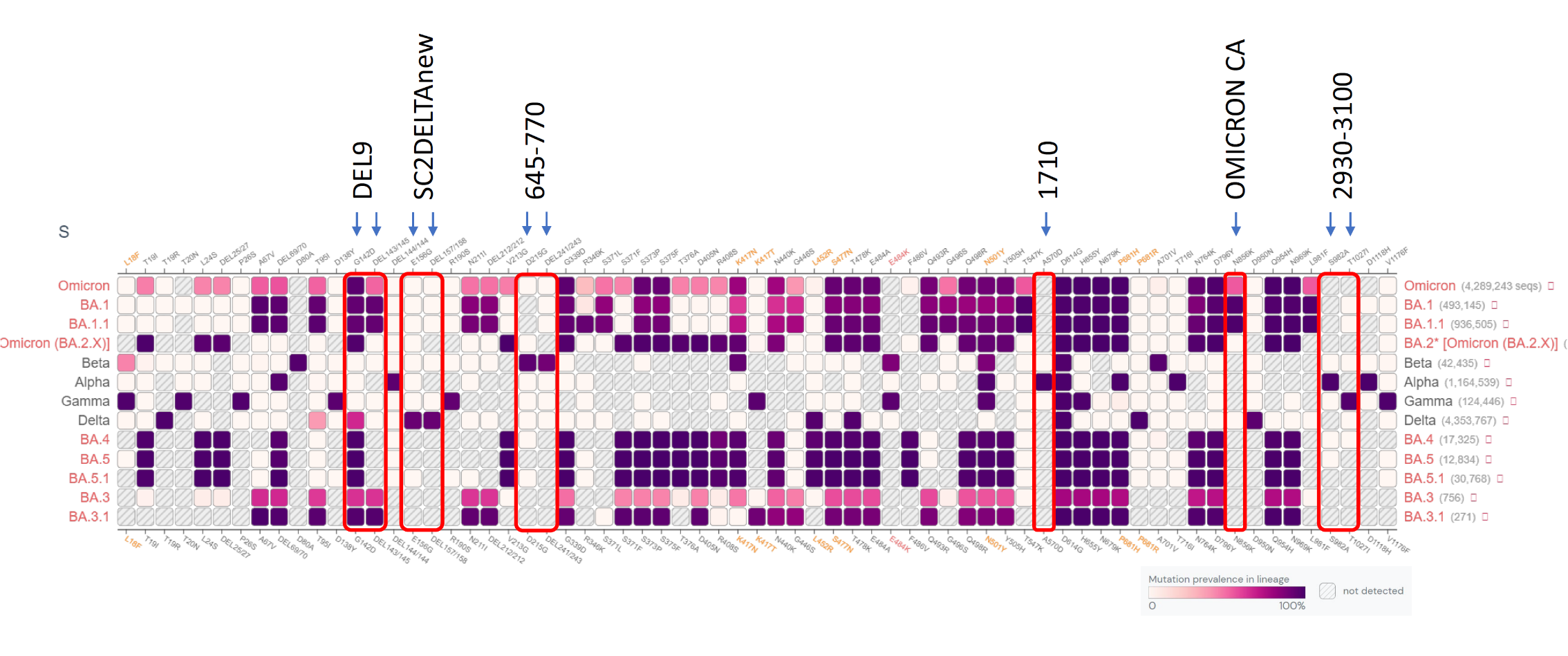

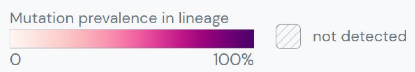


**Supplementary figure 1**. Mutation prevalence on the S gene across lineages, obtained from outbreak.info (https://outbreak.info/compare-lineages) (database accessed on July 13^th^, 2022). Mutations with > 75% prevalence in at least one lineage are shown. Lineages with fewer than 200 sequences have been removed. Mutations selected for RT-qPCR assays are boxed and indicated by arrows. Color intensities indicate mutation prevalence (from 0 to 100%); dashed squares indicate no mutation detected.


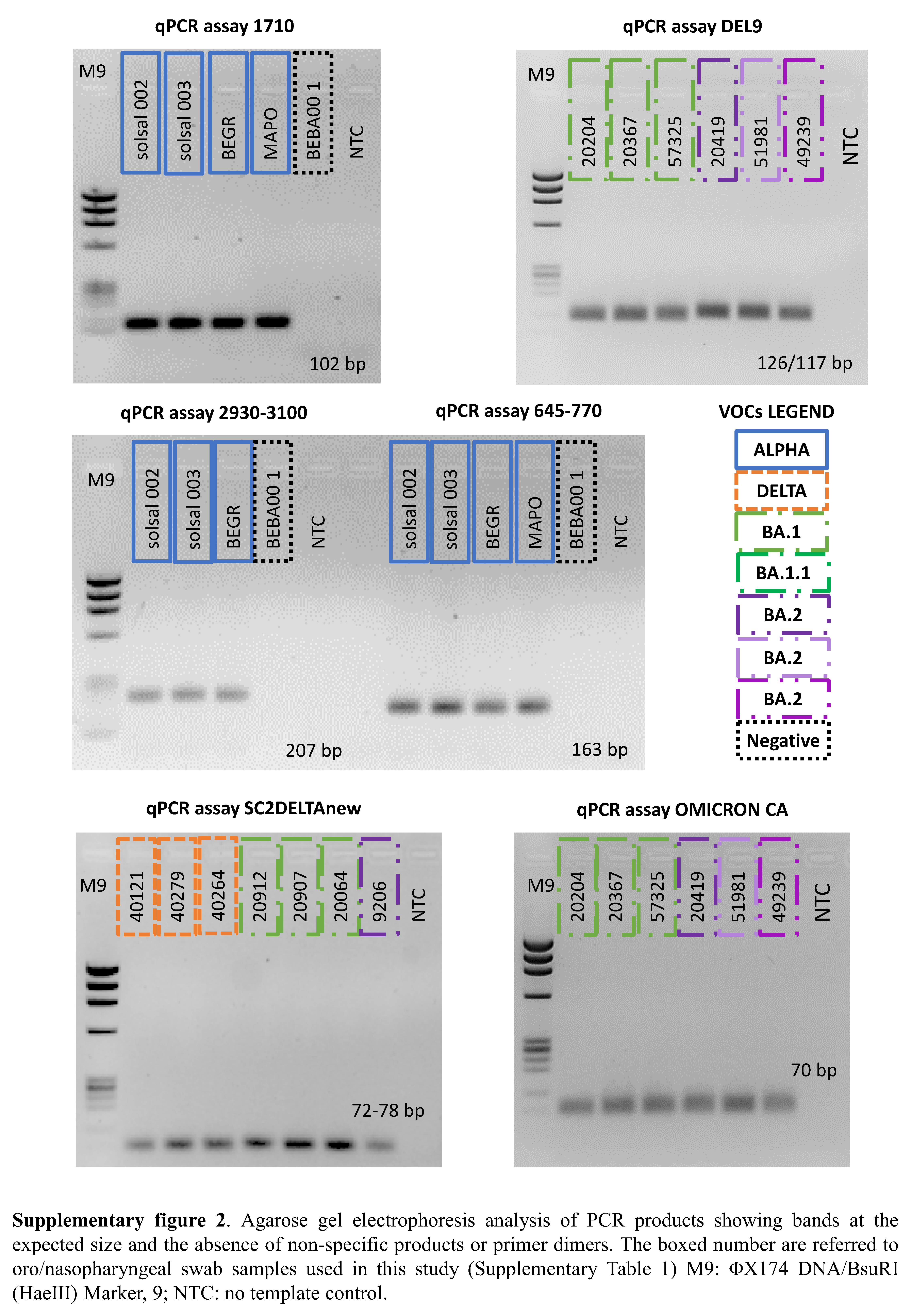


**Supplementary figure 2**. Agarose gel electrophoresis analysis of PCR products showing bands at the expected size and the absence of non-specific products or primer dimers. The boxed numbers are referred to oro/nasopharyngeal swab samples used in this study (Supplementary Table 1) M9: ΦX174 DNA/BsuRI (HaeIII) Marker, 9; NTC: no template control.


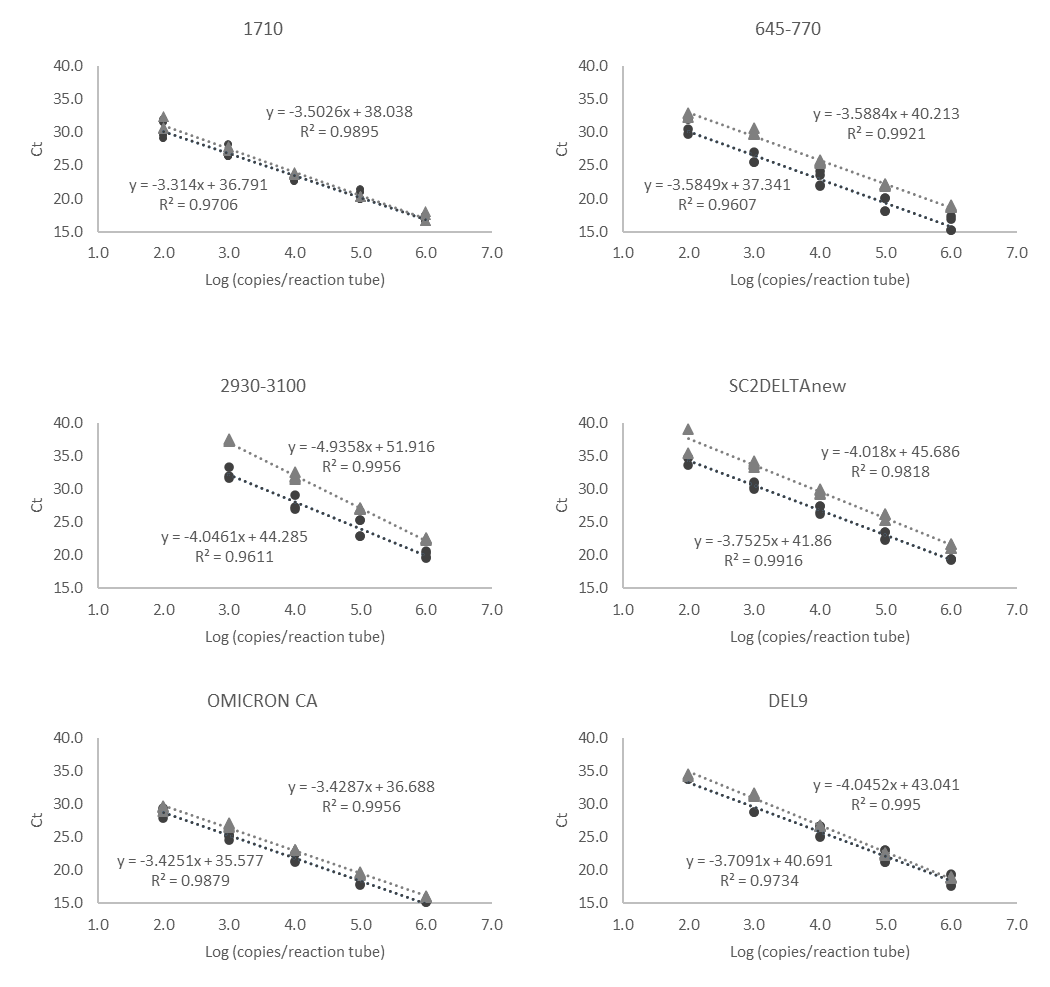


**Supplementary figure 3**. Analytical sensitivity of all qPCR assays used in this work tested using SARS-CoV2 synthetic RNA of the reference isolate Wuhan-hu-1. The equation of trendline and R^2^ values are indicated in the figure panels.


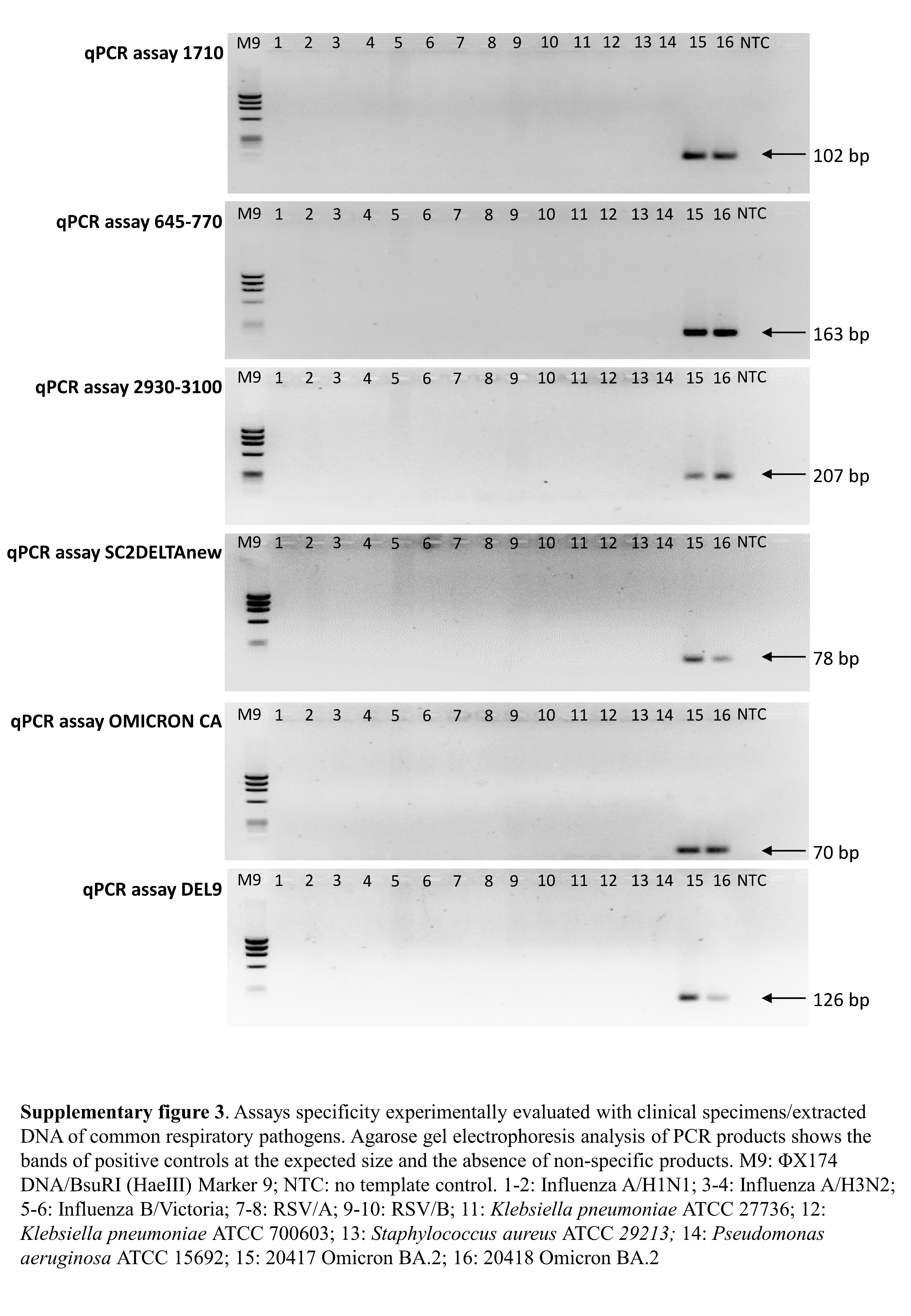


**Supplementary figure 4**. Assays specificity experimentally evaluated with clinical specimens/extracted DNA of common respiratory pathogens. Agarose gel electrophoresis analysis of PCR products shows the bands of positive controls at the expected size and the absence of non-specific products. M9: ΦX174 DNA/BsuRI (HaeIII) Marker 9; NTC: no template control. 1-2: Influenza A/H1N1; 3-4: Influenza A/H3N2; 5-6: Influenza B/Victoria; 7-8: RSV/A; 9-10: RSV/B; 11: *Klebsiella pneumoniae* ATCC 27736; 12: *Klebsiella pneumoniae* ATCC 700603; 13: *Staphylococcus aureus* ATCC *29213;* 14: *Pseudomonas aeruginosa* ATCC 15692; 15: 20417 Omicron BA.2; 16: 20418 Omicron BA.2


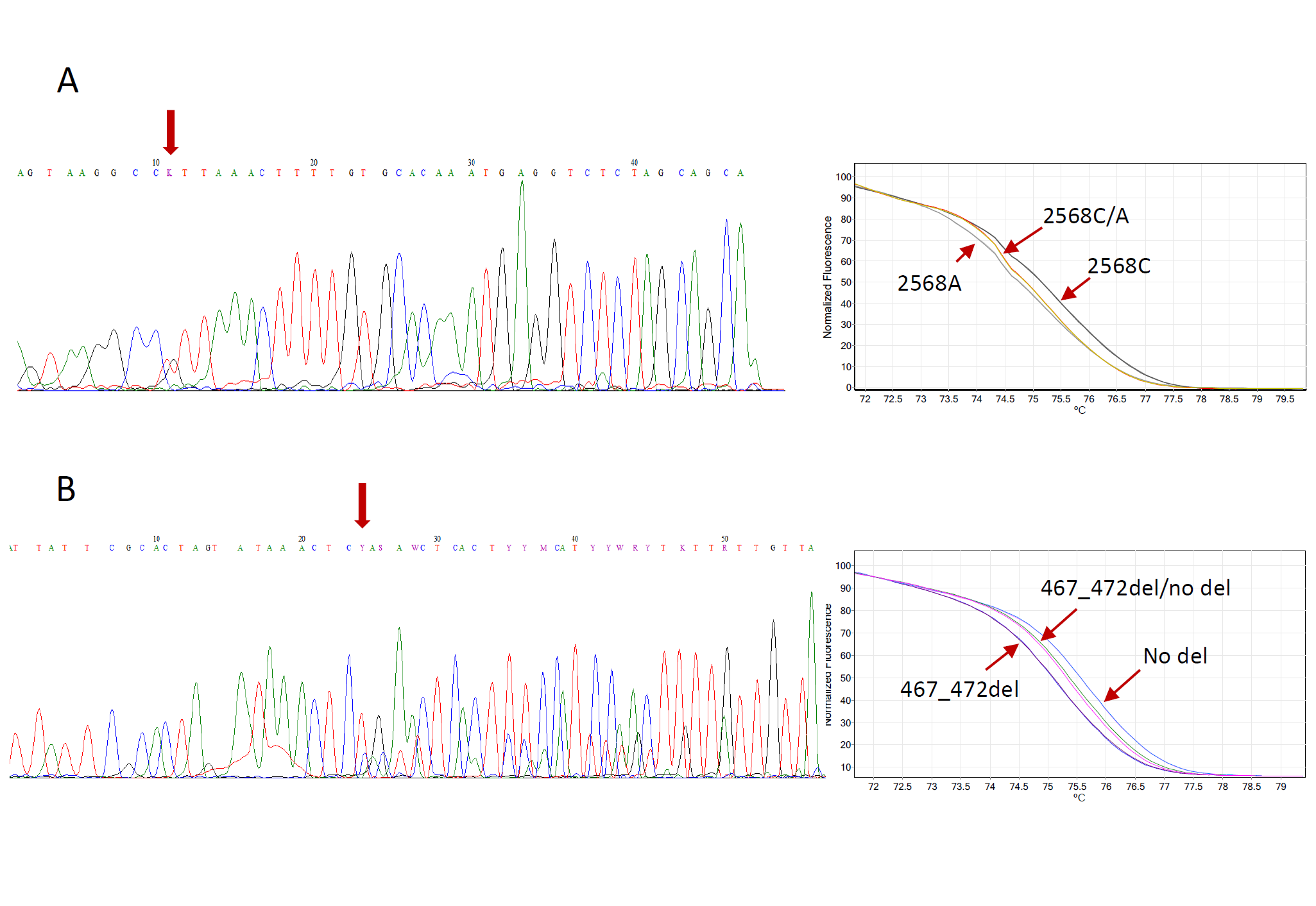


**Supplementary figure 5.** Electropherograms (left) and HRM analysis (right) of amplicons obtained from sample 20044. A) qPCR assay OMICRON CA; B) qPCR assay SC2DELTAnew.

**Supplementary Table 1.** Amplification results of clinical specimens positive for respiratory viruses

| **Sample** | **Target Name** | **Reporter** | **Ct** |
| --- | --- | --- | --- |
| Influenza A/H1N1  Sample 1 | Flu A/B  RSV A/B  SARS-CoV-2  RNAseP | VIC  ROX  FAM  CY5 | 24.81  Undetermined  Undetermined  27.22 |
| Influenza A/H1N1  Sample 2 | Flu A/B  RSV A/B  SARS-CoV-2  RNAseP | VIC  ROX  FAM  CY5 | 28.27  Undetermined  Undetermined  20.21 |
| Influenza A/H3N2 Sample 1 | Flu A/B  RSV A/B  SARS-CoV-2  RNAseP | VIC  ROX  FAM  CY5 | 27.44  Undetermined  Undetermined  23.03 |
| Influenza A/H3N2 Sample 2 | Flu A/B  RSV A/B  SARS-CoV-2  RNAseP | VIC  ROX  FAM  CY5 | 27.96  Undetermined  Undetermined  29.19 |
| Influenza B/Victoria  Sample 1 | Flu A/B  RSV A/B  SARS-CoV-2  RNAseP | VIC  ROX  FAM  CY5 | 26.09  Undetermined  Undetermined  20.52 |
| Influenza B/Victoria  Sample 2 | Flu A/B  RSV A/B  SARS-CoV-2  RNAseP | VIC  ROX  FAM  CY5 | 35.31  Undetermined  Undetermined  33.44 |
| RSV/A  Sample 1 | Flu A/B  RSV A/B  SARS-CoV-2  RNAseP | VIC  ROX  FAM  CY5 | Undetermined  21.49  Undetermined  29.62 |
| RSV/A  Sample 2 | Flu A/B  RSV A/B  SARS-CoV-2  RNAseP | VIC  ROX  FAM  CY5 | Undetermined  21.44  Undetermined  22.59 |
| RSV/B  Sample 1 | Flu A/B  RSV A/B  SARS-CoV-2  RNAseP | VIC  ROX  FAM  CY5 | Undetermined  18.83  Undetermined  21.19 |
| RSV/B  Sample 2 | Flu A/B  RSV A/B  SARS-CoV-2  RNAseP | VIC  ROX  FAM  CY5 | Undetermined  19.25  Undetermined  23.73 |
| PCR Positive control COVID-19, FLU-RSV | Flu A/B  RSV A/B  SARS-CoV-2  RNAseP | VIC  ROX  FAM  CY5 | 27.19  26.99  25.20  23.30 |

**Supplementary Table 2.** Summary of oro/nasopharyngeal swab samples used in this study and monitoring results

| **Sample number** | **Sample ID** | **Sampling date** | **VOC**  **(RT-qPCR)** | **VOC (sequencing)** |
| --- | --- | --- | --- | --- |
| 1 | solsal 002 | 16/04/2021 | Alpha | Alpha^b^ |
| 2 | solsal 003 | 16/04/2021 | Alpha | Alpha^b^ |
| 3 | BEGR | 28/04/2021 | Alpha | Alpha^b^ |
| 4 | MAPO | 05/05/2021 | Alpha | Alpha^b^ |
| 5 | BEBA001^a^ | 21/05/2021 | NEG | - |
| 6 | AF003 | 16/10/2020 | Wuhan* | Wuhan^b^ |
| 7 | 00000 | 19/10/2020 | Wuhan* | Wuhan^b^ |
| 8 | 78 | 19/10/2020 | Wuhan* | Wuhan^b^ |
| 9 | 234 | 19/10/2020 | Wuhan* | Wuhan^b^ |
| 10 | 99999 | 21/10/2020 | Wuhan* | Wuhan^b^ |
| 11 | VT00233 | 26/10/2020 | Wuhan* | Wuhan^b^ |
| 12 | VT00262 | 26/10/2020 | Wuhan* | Wuhan^b^ |
| 13 | VT00265 | 26/10/2020 | Wuhan* | Wuhan^b^ |
| 14 | IDRO-02 | 08/04/2021 | Alpha | Alpha^b^ |
| 15 | IDRO-03 | 08/04/2021 | Alpha | Alpha^b^ |
| 16 | IDRO-06 | 08/04/2021 | Alpha | Alpha^b^ |
| 17 | IDRO-07 | 08/04/2021 | Alpha | Alpha^b^ |
| 18 | IDRO-13 | 08/04/2021 | Alpha | Alpha^b^ |
| 19 | FU001AG | 08/04/2021 | Alpha | Alpha^b^ |
| 20 | FU002MF | 08/04/2021 | Alpha | Alpha^b^ |
| 21 | BA-6150 | 03/06/2021 | Alpha | Alpha^b^ |
| 22 | UULFFH70XV | 28/08/2021 | Delta | n.a. |
| 23 | UULFFH72XV | 28/08/2021 | Delta | n.a. |
| 24 | UULFHMJUXV | 28/08/2021 | Delta | Delta^b^ |
| 25 | UULFHMKXXV | 28/08/2021 | Delta | Delta^b^ |
| 26 | UULGZ7JTXV | 18/09/2021 | Delta | Delta^b^ |
| 27 | UULGZ7JSXV | 18/09/2021 | Delta | Delta^b^ |
| 28 | UULGZ7K9XV | 18/09/2021 | Delta | n.a. |
| 29 | UULGZ7K4XV | 18/09/2021 | Delta | n.a. |
| 30 | 20542/07 | 07/01/2022 | Delta | Delta^c^ |
| 31 | 9205/25 | 24/12/2021 | Delta | Delta^c^ |
| 32 | 2163/28 | 28/12/2021 | BA.1/BA.1.1 | BA.1^c^ |
| 33 | 3109/11 | 12/01/2022 | BA.1/BA.1.1 | BA.1^c^ |
| 34 | 20055/25 | 21/12/2021 | Delta | Delta^c^ |
| 35 | 20542/09 | 20/12/2021 | Delta | Delta^c^ |
| 36 | 20440/04 | 04/01/2022 | BA.1/BA.1.1 | BA.1^c^ |
| 37 | 9216/09 | 09/01/2022 | BA.1/BA.1.1 | BA.1^c^ |
| 38 | 20624/24 | 24/12/2021 | BA.1/BA.1.1 | BA.1^c^ |
| 39 | 9206/06 | 06/01/2022 | BA.1/BA.1.1 | BA.1^c^ |
| 40 | 9219/05 | 05/01/2022 | BA.1/BA.1.1 | BA.1^c^ |
| 41 | 2033/08 | 08/01/2022 | BA.1/BA.1.1 | BA.1^c^ |
| 42 | 9204/28 | 28/12/2021 | BA.1/BA.1.1 | BA.1^c^ |
| 43 | 20505/07 | 07/01/2022 | BA.1/BA.1.1 | BA.1^c^ |
| 44 | 40121 | 11/08/2021 | Delta | Delta^c^ |
| 45 | 40279 | 19/08/2021 | Delta | Delta^c^ |
| 46 | 40264 | 16/09/2021 | Delta | Delta^c^ |
| 47 | 20912/3 | 03/01/2022 | BA.1/BA.1.1 | BA.1^c^ |
| 48 | 20907/3 | 03/01/2022 | BA.1/BA.1.1 | BA.1^c^ |
| 49 | 20064/1 | 01/01/2022 | BA.1/BA.1.1 | BA.1^c^ |
| 50 | 9206/6 | 06/02/2022 | BA.2 ^#^ | BA.2^c^ |
| 51 | 71000 | 23/07/2021 | Delta | Delta^c^ |
| 52 | 40201 | 10/09/2021 | Delta | Delta^c^ |
| 53 | 40113 | 25/10/2021 | Delta | Delta^c^ |
| 54 | 9218 | 08/09/2021 | Delta | Delta^c^ |
| 55 | 20547/17 | 17/01/2022 | BA.1/BA.1.1 | BA.1^c^ |
| 56 | 20629/18 | 18/01/2022 | BA.1/BA.1.1 | BA.1^c^ |
| 57 | 20315/10 | 10/02/2022 | BA.1/BA.1.1 | BA.1^c^ |
| 58 | 20346/10 | 10/02/2022 | BA.1/BA.1.1 | BA.1^c^ |
| 59 | 40094 | 28/08/2021 | Delta | Delta^c^ |
| 60 | 20021 | 11/02/2022 | BA.1/BA.1.1 | BA.1^c^ |
| 61 | 20044 | 19/12/2021 | Not assigned | Delta/ BA.1^b^ |
| 62 | 24 | 30/12/2021 | BA.1/BA.1.1 | n.a. |
| 63 | 20238 | 03/01/2022 | BA.1/BA.1.1 | n.a. |
| 64 | 9222 | 03/01/2022 | BA.1/BA.1.1 | n.a. |
| 65 | 20096 | 13/01/2022 | BA.1/BA.1.1 | n.a. |
| 66 | 20049 | 13/01/2022 | BA.1/BA.1.1 | n.a. |
| 67 | 20205 | 14/02/2022 | BA.1/BA.1.1 | n.a. |
| 68 | 20222 | 17/02/2022 | BA.1/BA.1.1 | n.a. |
| 69 | 20800 | 20/12/2021 | Delta | n.a. |
| 70 | 20444 | 20/12/2021 | Delta | n.a. |
| 71 | 20208 | 03/01/2022 | BA.1/BA.1.1 | n.a. |
| 72 | 20781^a^ | 03/01/2022 | NEG | - |
| 73 | 20465 | 08/01/2022 | BA.1/BA.1.1 | n.a. |
| 74 | 20093 | 13/01/2022 | BA.1/BA.1.1 | n.a. |
| 75 | 5510^a^ | 14/01/2022 | NEG | - |
| 76 | 20058 | 14/01/2022 | BA.1/BA.1.1 | n.a. |
| 77 | 20534^a^ | 17/01/2022 | NEG | - |
| 78 | 20287 | 05/02/2022 | BA.1/BA.1.1 | n.a. |
| 79 | 20444 | 09/02/2022 | BA.1/BA.1.1 | n.a. |
| 80 | 20129 | 15/02/2022 | BA.1/BA.1.1 | n.a. |
| 81 | 20457 | 17/03/2022 | BA.2^#^ | BA.2^c^ |
| 82 | 20369 | 7/03/2022 | BA.1/BA.1.1 | BA.1.1^c^ |
| 83 | 20394 | 7/03/2022 | BA.1/BA.1.1 | BA.1^c^ |
| 84 | 20317 | 23/12/2021 | Delta | Delta^b^ |
| 85 | 20391 | 14/01/2022 | BA.1/BA.1.1 | BA.1/BA.1.1^b^ |
| 86 | 20186 | 07/02/2022 | BA.1/BA.1.1 | BA.1/BA.1.1^b^ |
| 87 | 20573 | 15/02/2022 | BA.1/BA.1.1 | BA.1/BA.1.1^b^ |
| 88 | 20575 | 15/02/2022 | BA.1/BA.1.1 | BA.1/BA.1.1^b^ |
| 89 | 57736 | 05/03/2022 | BA.1/BA.1.1 | BA.1^c^ |
| 90 | 57326 | 07/03/2022 | BA.1/BA.1.1 | BA.1.1^c^ |
| 91 | 53834 | 07/03/2022 | BA.2/BA.4/BA.5 | BA.2^c^ |
| 92 | 52467 | 05/06/2022 | BA.2/BA.4/BA.5 | BA.4 ^c^ |
| 93 | 48710 | 07/06/2022 | BA.2/BA.4/BA.5 | BA.5^c^ |
| 94 | 20204 | 18/03/2022 | BA.1/BA.1.1 | BA.1^c^ |
| 95 | 56007 | 07/03/2022 | BA.1/BA.1.1 | BA.1^c^ |
| 96 | 54841 | 07/03/2022 | BA.1/BA.1.1 | BA.1^c^ |
| 97 | 20367 | 07/03/2022 | BA.1/BA.1.1 | BA.1.1^c^ |
| 98 | 57325 | 07/03/2022 | BA.1/BA.1.1 | BA.1.1^c^ |
| 99 | 51346 | 07/03/2022 | BA.1/BA.1.1 | BA.1.1^c^ |
| 100 | 20417 | 23/02/2022 | BA.2/BA.4/BA.5 | BA.2^c^ |
| 101 | 20418 | 23/02/2022 | BA.2/BA.4/BA.5 | BA.2^c^ |
| 102 | 20419 | 23/02/2022 | BA.2/BA.4/BA.5 | BA.2^c^ |
| 103 | 20391 | 03/03/2022 | BA.2/BA.4/BA.5 | BA.2^c^ |
| 104 | 20084 | 05/03/2022 | BA.2/BA.4/BA.5 | BA.2^c^ |
| 105 | 56008 | 07/03/2022 | BA.2/BA.4/BA.5 | BA.2^c^ |
| 106 | 53885 | 07/03/2022 | BA.2/BA.4/BA.5 | BA.2^c^ |
| 107 | 49095 | 08/06/2022 | BA.1/BA.1.1 | BA.4 ^c^ |
| 108 | 51981 | 09/06/2022 | BA.2/BA.4/BA.5 | BA.4 ^c^ |
| 109 | 49239 | 07/06/2022 | BA.2/BA.4/BA.5 | BA.5^c^ |
| 110 | 55041 | 11/06/2022 | BA.2/BA.4/BA.5 | BA.5^c^ |
| 111 | uniurb 01^a^ | 07/10/2021 | NEG | - |
| 112 | uniurb 02^a^ | 07/10/2021 | NEG | - |
| 113 | uniurb 03^a^ | 07/10/2021 | NEG | - |
| 114 | uniurb 04^a^ | 07/10/2021 | NEG | - |
| 115 | uniurb 05^a^ | 07/10/2021 | NEG | - |
| 116 | uniurb 06^a^ | 07/10/2021 | NEG | - |
| 117 | uniurb 07^a^ | 07/10/2021 | NEG | - |
| 118 | uniurb 08^a^ | 07/10/2021 | NEG | - |
| 119 | uniurb 01^a^ | 09/12/2021 | NEG | - |
| 120 | uniurb 02^a^ | 09/12/2021 | NEG | - |

^a^ negative sample

^b^ PCR product sequencing

^c^ WGS

^*^Assigned to Wuhan since other VOCs were not present in the referred period

^#^ Assigned to BA.2 since BA.4 and BA.5 were not present in the referred period
